# High correlation between genotypes and phenotypes of environmental bacteria *Comamonas testosteroni* strains

**DOI:** 10.1186/s12864-015-1314-x

**Published:** 2015-02-21

**Authors:** Lin Liu, Wentao Zhu, Zhan Cao, Biao Xu, Gejiao Wang, Meizhong Luo

**Affiliations:** State Key Laboratory of Agricultural Microbiology and College of Life Science and Technology, Huazhong Agricultural University, Wuhan, Hubei 430070 People’s Republic of China

**Keywords:** Comamonas testosteroni, Comparative genomics, Genotype, Phenotype

## Abstract

**Background:**

Members of *Comamonas testosteroni* are environmental microorganisms that are usually found in polluted environment samples. They utilize steroids and aromatic compounds but rarely sugars, and show resistance to multiple heavy metals and multiple drugs. However, comprehensive genomic analysis among the *C. testosteroni* strains is lacked.

**Results:**

To understand the genome bases of the features of *C. testosteroni*, we sequenced 10 strains of this species and analyzed them together with other related published genome sequences. The results revealed that: 1) the strains of *C. testosteroni* have genome sizes ranging from 5.1 to 6.0 Mb and G + C contents ranging from 61.1% to 61.8%. The pan-genome contained 10,165 gene families and the core genome contained 3,599 gene families. Heap’s law analysis indicated that the pan-genome of *C. testosteroni* may be open (*α* = 0.639); 2) by analyzing 31 phenotypes of 11 available *C. testosteroni* strains, 99.4% of the genotypes (putative genes) were found to be correlated to the phenotypes, indicating a high correlation between phenotypes and genotypes; 3) gene clusters for nitrate reduction, steroids degradation and metal and multi-drug resistance were found and were highly conserved among all the genomes of this species; 4) the genome similarity of *C. testosteroni* may be related to the geographical distances*.*

**Conclusions:**

This work provided an overview on the genomes of *C. testosteroni* and new genome resources that would accelerate the further investigations of this species. Importantly, this work focused on the analysis of potential genetic determinants for the typical characters and found high correlation between the phenotypes and their corresponding genotypes.

**Electronic supplementary material:**

The online version of this article (doi:10.1186/s12864-015-1314-x) contains supplementary material, which is available to authorized users.

## Background

*Comamonas testosteroni* is a bacterial species belonging to β*-*Proteobacteria which was originally named as *Pseudomonas testosteroni* using a soil bacterium ATCC 11996 as the type strain [1]. In 1987, Tamaoka *et al*. reclassified this species into *Comamonas testosteroni* based on phylogenetic analysis [2]. *C. testosteroni* strains are capable of utilizing testosterone, 4-hydroxybenzoate (4HBA), acetate and lactate as their sole carbon sources, but not glucose and most of the carbohydrates [3]. They are strictly aerobic, highly motile and gram-negative rods and can perform nitrate reduction but cannot denitrify [3].

*C. testosteroni* strains have been paid a great attention duo to their characteristics of degradation for pollutants and resistance to heavy metals. For example, most strains of *C. testosteroni* were isolated from such environments as active sludge [4,5] and heavy metal-contaminated mining soil [6]; *C. testosteroni* T-2 could utilize 4-toluenesulphonic acid and 4-sulphobenzoic acid [7]; *C. testosteroni* SPB-2 (KF-1) could utilize 4-sulfophenylcarboxylates [8]. *C. testosteroni* CNB-1 have successfully been applied in environment bioremediation to degrade 4-chloronitrobenzene (4CNB) in the soil [9].

The genetic determinants for the organic compound degradation and heavy metal resistance have been analyzed in several *C. testosteroni* strains. Horinouchi *et al*. discovered five gene clusters for steroid degradation in the genome of *C. testosteroni* TA441 [10-15]. Gene operons responsible for degrading isophthalate and terephthalate to protocatechuate were determined in *C. testosteroni* YZW-D by Wang *et al.* [16]. The operon *aph* (KLMNOPQB) that encodes phenol hydroxylase and catechol 2,3-dioxygenase, and the operon *aph* (CEFGHJI) that encodes the enzymes for degrading 2-hydroxymuconic semialdehyde to TCA cycle intermediates were found in *C. testosteroni* TA441 [17,18]. A gene cluster for protocatechuate (PCA) 4,5-cleavage pathway, which is an important pathway for aromatic compounds, such as 4HBA degradation [19], were found in the genome of *C. testosteroni* CNB-2 [20]. *C. testosteroni* CNB-1 showed resistance to arsenate and an *ars* (RPBC) operon was found on the pCNB1 plasmid [5,21]. Xiong *et al.* [6] have reported ZntA genes responsible for Zn resistance in *C. testosteroni* S44.

So far, the genomic sequences of four *C. testosteroni* strains have been published. Ma *et al.* [5] sequenced the whole genome of *C. testosteroni* CNB-2, analyzed its general genome features, and found genes for many functions such as aromatic compound metabolism, transportation system and drug and heavy metal resistance. Gong *et al.* [22] sequenced the genome of the type strain of *C. testosteroni* ATCC 11996. Weiss *et al.* [23] sequenced the genome of strain KF-1, and found genes for degradation of aromatic compounds such as a gene cluster for PCA 4,5-cleavage and a gene cluster for isophthalate degradation. In our previous study, a multiple heavy-metal resistant strain named *C. testosteroni* S44 was isolated and its genome was sequenced [6]. A series of metal-resistant genes and *g*ene clusters for aromatic compound degradation were found in its genome [6,24]. Regarding carbohydrates utilization, studies on both genomes of *C. testosteroni* strains CNB-2 and ATCC 11996 showed that the genes encoding hexokinase and glucokinase were missing [5,22]. However, a comprehensive genomic analysis among the *C. testosteroni* strains is lacked.

The objective of this study was to analyze the genetic basis responsible for important biological characters in multiple *C. testosteroni* strains. Recently, we have identified ten *C. testosteroni* strains from different mining soils [24,25] according to the 16S rRNA gene and physiological and biochemical analysis*.* In order to gain a comprehensive genotypic and phonotypic understanding, we sequenced the ten *C. testosteroni* strains and analyzed them together with the four published *C. testosteroni* genomes [5,6,22,23]. This study is a first comparative genomic analysis for *C. testosteroni* strains. The results have revealed information to better understand this bacterial species at genomic level, and importantly, have pointed out potential genetic determinants for the typical characters and high correlation between the phenotypes and their corresponding genotypes (genes).

## Methods

### Genome analysis of *C. testosteroni* and related strains

A total of 14 *C. testosteroni* strains were used for genomic analysis. Among them, *C. testosteroni* strains JC8, JC9, JC12, JC13, DF1, DF2, DS1 [25], JL14, JL40 [24] and D4 were isolated from different mining soils in China (Additional file 1: Table S1) and sequenced in this study by Majorbio Biomedical Science and Technology Co. Ltd using Illumina Hiseq2000 and assembled by SOAPdenovo v1.05 [26]. The draft sequences were submitted to GenBank Whole Genome Shotgun (WGS) database [27] and the accession numbers are shown in Table 1. Genome sequences of four other *C. testoteroni* strains, CNB-2 [5], ATCC 11996 [22], KF-1 [23] and S44 [6] (Table 1) were obtained from the NCBI database. Genome sequences of eight strains of the *Comamonadaceae* family were also obtained from the NCBI database (Additional file 2: Table S2). They are *Acidovorax avenae* subsp. *avenae* RS-1 [28], *Alicycliphilus denitrificans* K601 [29], *Delftia acidovorans* SPH-1 [8], *Polaromonas naphthalenivorans* CJ2 [30], *Ramlibacter tataouinensis* TTB310 [31], *Hylemonella gracilis* ATCC 19624 [32], *Variovorax paradoxus* EPS [33], and *Verminephrobacter aporrectodeae* subsp. *tuberculatae* At4 [34]. Annotation/re-annotation of all the genome sequences was performed on-line by Prokaryotic Genome Annotation Server (RAST) [35-37]. The annotation results are available at RAST server (http://rast.nmpdr.org) with user ID: C_t_visitor and password: testosteroni.

**Table 1:** **General features of the genomes of the** ***Comamonas testosteroni*** **strains used in this study**

**Strain**	**Source**	**Size (Mb)**	**GC (%)**	**No. of ORFs**	**Avg. ORF length (bp)**	**Coding density(%)**	**Accession number (reference)**
*C. testosteroni* JC8	Coal mine soil	5.36	61.1	4,935	934	86.04	AWOS00000000 (this study)
*C. testosteroni* JC9	Coal mine soil	5.37	61.1	4,957	934	86.15	AWOT00000000 (this study)
*C. testosteroni* JC12	Coal mine soil	5.36	61.1	4,948	932	86.06	AWOU00000000 (this study)
*C. testosteroni* JC13	Coal mine soil	5.33	61.2	4,914	934	86.20	AWOV00000000 (this study)
*C. testosteroni* JL14	Sb mine soil	5.75	61.2	5,416	900	84.88	AWTN00000000 (this study)
*C. testosteroni* JL40	Sb mine soil	5.96	61.1	5,546	914	85.12	AWOR00000000 (this study)
*C. testosteroni* D4	As polluted soil	5.06	61.6	4,674	938	86.62	AWTQ00000000 (this study)
*C. testosteroni* DS1	Cu-Fe mine soil	5.69	61.1	5,400	897	85.22	AWTM00000000 (this study)
*C. testosteroni* DF1	Fe mine soil	5.59	61.1	5,280	906	85.62	AWTO00000000 (this study)
*C. testosteroni* DF2	Fe mine soil	5.61	61.1	5,294	907	85.58	AWTP00000000 (this study)
*C. testosteroni* S44	Sb mine soil	5.53	61.4	5,147	921	85.70	ADVQ01000000 [6]
*C. testosteroni* CNB-2	Active sludge	5.37	61.4	5,011	924	86.15	NC_013446 [5]
*C. testosteroni* ATCC 11996	Soil	5.41	61.5	4,998	931	86.05	AHIL01000000 [22]
*C. testosteroni* KF-1	Active sludge	6.03	61.8	5,645	920	86.11	AAUJ02000000 [23]

### Ortholog clustering analysis

The OrthoMCL package [38] was used to determine the core-genome and unique genes of each genome. All predicted protein sequences were grouped together and compared with each other using blastp of NCBI-blast 2.27+ program [39]. The homologous protein pairs with E-value cutoff 1e-5 and percent of match ≥ 50% were parsed, and the normalized homologous scores were calculated. We screened the homologous protein pairs by a custom Perl script with percent of identity greater than 50%. Then the proteins were grouped into orthologous families by cluster tool MCL (MCL; Van Dongen 2000; http://micans.org/mcl/), with the inflation value of 1.5. The unique genes of each genome were identified based on the resultant orthologous families by a custom Perl script. The core-genome sizes were estimated by summing the orthologous families that contained genes from all the selected genomes. The pan-genome sizes were estimated by summing all the orthologous families and the single genes together. Heap’s law [40] was used to calculate the constant *α* in power law regression analysis *n* = *κN*^‐ *a*^ which could determine whether pan-genome is open (α ≤ 1) or closed (α > 1) (where *n* is the number of new genes, *N* is the number of genomes, κ is another constant).

### Phylogenetic analysis

Consulting the method of Li *et al.* [41] and Collins *et al.* [42], the single copy orthologous gene families (contain one gene from one strain), which were extracted from the genomes of 14 *C. testosteroni* strains and from the genomes of 22 strains of family *Comamonadaceae*, were used to build phylogenomic trees to present the phylogenetic relationship. The predicted amino acid sequences of each gene family were aligned using Clustal W [43]. The independent alignments were concatenated to form a pseudo amino acid sequence alignment. The result was submitted to MEGA6 [44] to build Neighbor-joining (NJ) trees with p-distance. The bootstrap method of 1,000 bootstrap repetitions was used to assess tree reliability. Average Nucleotide Identity (ANI) analysis was performed among the 14 genomes from different sampling areas using Jspecies1.2.1 [45].

### Analysis of important physiological and biochemical characteristics of 11 *C. testosteroni* strains

Most of physiological and biochemical characteristics were determined using the API 20NE system (bioMe’rieux, Marcyl’Etoile, France), except for the utilization of citrate that was detected using Simmons’ citrate assimilation test [46]. For utilization of acetate, testosterone and 4HBA, the strains were cultured in basal medium containing standard minerals based on Stanier *et al.* [3] and with 0.3% (wt/vol) sodium acetate trihydrate, 0.2% (wt/vol) testosterone and 0.2% (wt/vol) sodium 4HBA, respectively, and incubated at 28°C for 10 days. The Minimum Inhibitory Concentrations (MICs) of Hg(II), Cu(II) and Zn(II) were detected in CDM solid medium [47] containing increasing concentrations of HgCl_2_, CuSO_4_•5H_2_O, and ZnCl_2_, respectively [48]. Antibiotic susceptibility tests for penicillins (penicillin, prostaphlin and ampicillin), kanamycin, and tetracycline were detected using disk diffusion test on Mueller-Hinton agar with 10 μg penicillin, 1 μg prostaphlin, 10 μg ampicillin, 30 μg kanamycin and 30 μg tetracycline disks, respectively (HangZhou Microbial Reagent Co. LTD, China).

### Construction of putative metabolic pathways based on the genomic sequences

The predicted proteins of each genome were submitted to KEGG Automatic Annotation Server (KAAS) [49] to perform KEGG Orthology (KO) functional annotation. The results were then submitted to Mapper of Kyoto Encyclopedia of Genes and Genomes (KEGG) [50] to reconstruct metabolic pathways.

### Identification of heavy metal- and drug-resistant genes and virulence factors

Genes involved in heavy metal- and drug-resistance were identified base on the SEED gene functional classification of the “resistance to antibiotics and toxic compounds” subsystem on RAST server [35-37]. The identified genes were grouped into gene clusters. Orthologous genes from each genome were aligned by Clustal W [43], concatenated, and then submitted to MEGA6 [44] to build NJ trees with p-distance and bootstrap method of 1000 bootstrap repetitions.

The virulence factors were identified by aligning the predicted protein sequences from the genomes of 14 *C. testosteroni* strains against the Virulence Factors Data Bases (VFDB) [51] using blastp of NCBI-blast 2.27+ with the standard of E-value ≤1e-5, identity ≥ 50%, match ≥ 70%.

### Identification of putative genes responsible for the physiological and biochemical characteristics

Genes for nitrate/nitrite reduction were identified base on the SEED gene functional classification of the “Nitrate and nitrite ammonification” subsystem on RAST server [35-37]. Five gene clusters for steroid degradation that were found in *C. testosteroni* strain TA441 (GenBank: AB489116, AB040808, AB063482, AB076368, AB186487) [10-15] were searched against each genome sequence using blastn of NCBI-blast2.27+ program [39]. Protein sequences from these 5 gene clusters were also searched against all the predicted proteins in the 22 genomes using blastp. Gene cluster of PCA 4,5-cleavage [20] that is potentially involved in 4HBA degradation was searched against the predicted proteins of genomes of *C. testosteroni* using blastp. The orthologous genes for steroid degradation, PCA 4,5-cleavage and nitrate/nitrite reduction were used to build evolutionary trees. Amino acid sequences of orthologous genes were aligned by Clustal W [43], concatenated, and submitted to MEGA6 [44] to build NJ trees with p-distance and bootstrap method of 1000 bootstrap repetitions. The correlation value between genotypes and phenotypes is calculated based on the phenotypic results compared with the presence or absence of the putative corresponding genes.

## Results

### Genome sequencing and features of 14 *C. testosteroni* strains

Ten genomes of *C. testosteroni* (strains JC8, JC9, JC12, JC13, JL14, JL40, D4, DS1, DF1 and DF2) were sequenced in this study using Illumina HiSeq2000. After trimming the raw data, we obtained 7,130,708 to 16,794,780 paired reads and 147,354 to 383,694 single reads with the average read lengths from 94 bp to 96 bp for each genome. The sequencing depths were from 116.2x to 283.2x. The reads of each genome were assembled into 75 to 238 contigs, and the total length of each draft genome was from 5,061,365 bp to 5,956,604 bp. The contig N50 was from 98,152 bp to 242,732 bp, and the contig N90 was from 18,558 bp to 56,606 bp. The details about the sequencing and assembly of each genome are showed in Additional file 3: Table S3.

Features of the ten newly sequenced and four published genomes of *C. testosteroni* were showed in Table 1. The sizes of *C. testosteroni* genomes are from 5.06 Mb to 6.03 Mb, and the G + C contents are from 61.1% to 61.8%. The predicted numbers of open reading frames (ORFs) are from 4674 to 5645 with the average lengths from 897 bp to 938 bp. These coding sequences cover 85% to 86% of their genome sequences.

### The core- and pan-genomes of *C. testosteroni*

**Figure 1:**
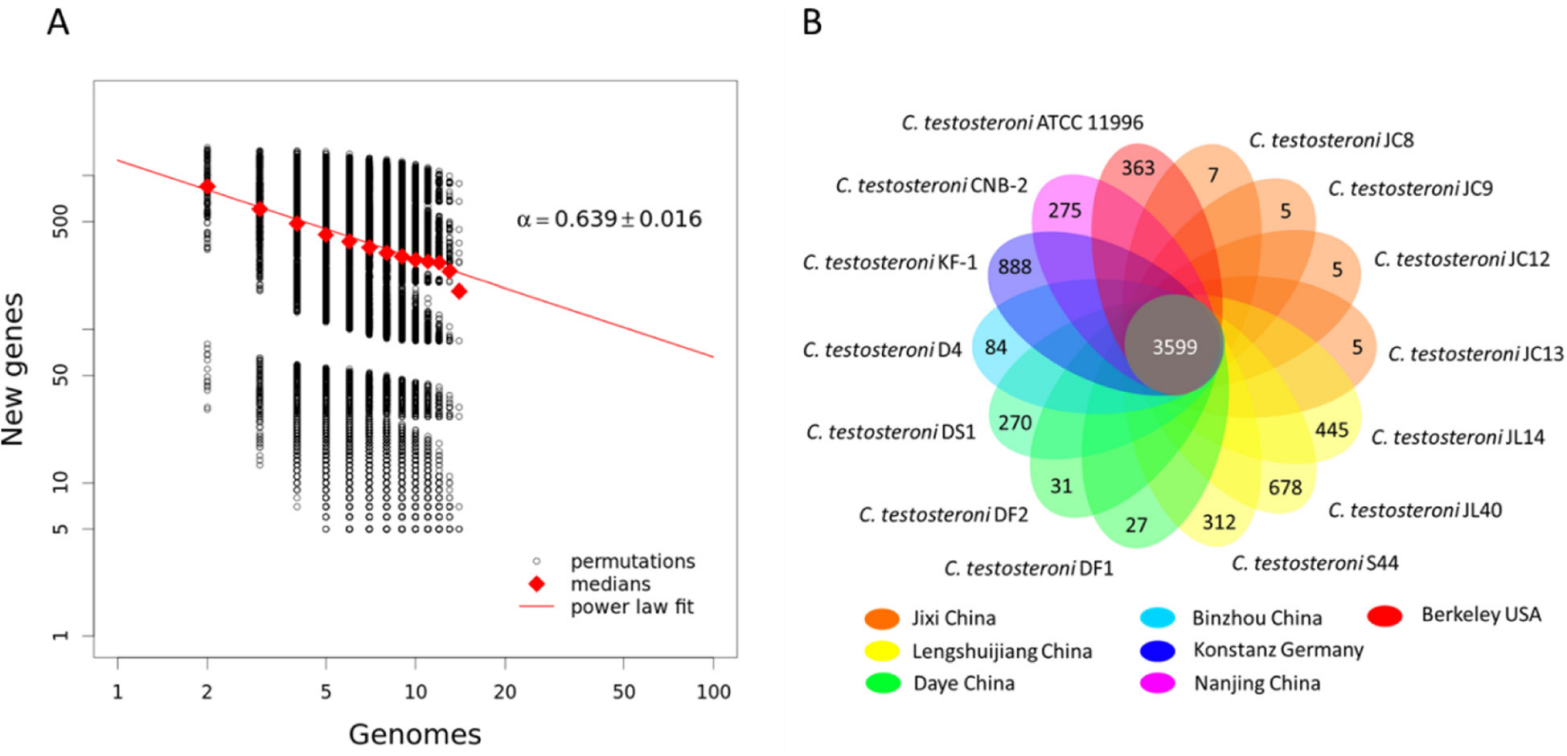
**Comparison among the 14 genomes of** ***C. testosteroni.*** **A)** Heap’s law [40] analysis to calculate the constant α. Power law regression was used to calculate the new genes in additional genomes of *C. testosteroni.* The dots indicated the number of new genes found according to Tettelin’s [58] method. The red curve is the least squares fit of the power law *n* = *κN* ^‐ *a*^ (κ = 1256 and α = 0.639) to medians of new genes (red diamond). **B)** The numbers of orthologous gene families and unique genes. The Venn diagram shows the number of orthologous gene families of the core-genome (the center part) and the numbers of unique genes of each genome. The different colors indicated different sampling areas of the strains as indicated. The orthologous gene families were determined by OrthoMCL software with the inflation value of 1.5. The unique genes of each genome are listed in Additional file 4: Table S4.

To determine whether the pan-genome of *C. testosteroni* is closed or open, the Heap’s law [40] was used in our study. As showed in Figure 1A, the medians of new genes (*n*) and the numbers of genomes (*N*) are least squares fit of the power law *n* = *κN*^‐ *a*^, and the constant α is 0.639 ± 0.016. This indicated that *C. testosteroni* may have an open pan-genome. For the 14 genomes, the core-genome has 3599 gene families, and the pan-genome has 10,165 gene families. The pan-genome size is two times of the average genome size and 2.8 times of the core-genome size. The core-genome accounts for 35% of the pan-genome and 64.7% to 77.8% of each individual genome. In each genome, the unique genes (listed in Additional file 4: Table S4) are from 5 to 888, accounting for 0.1% (JC12, JC13) to 15.7% (KF-1) of its total gene numbers (Figure 1B).

### Phylogenetic relationships between *C. testosteroni* and other species in family *Comamonadacea* and among *C. testosteroni* strains

**Figure 2:**
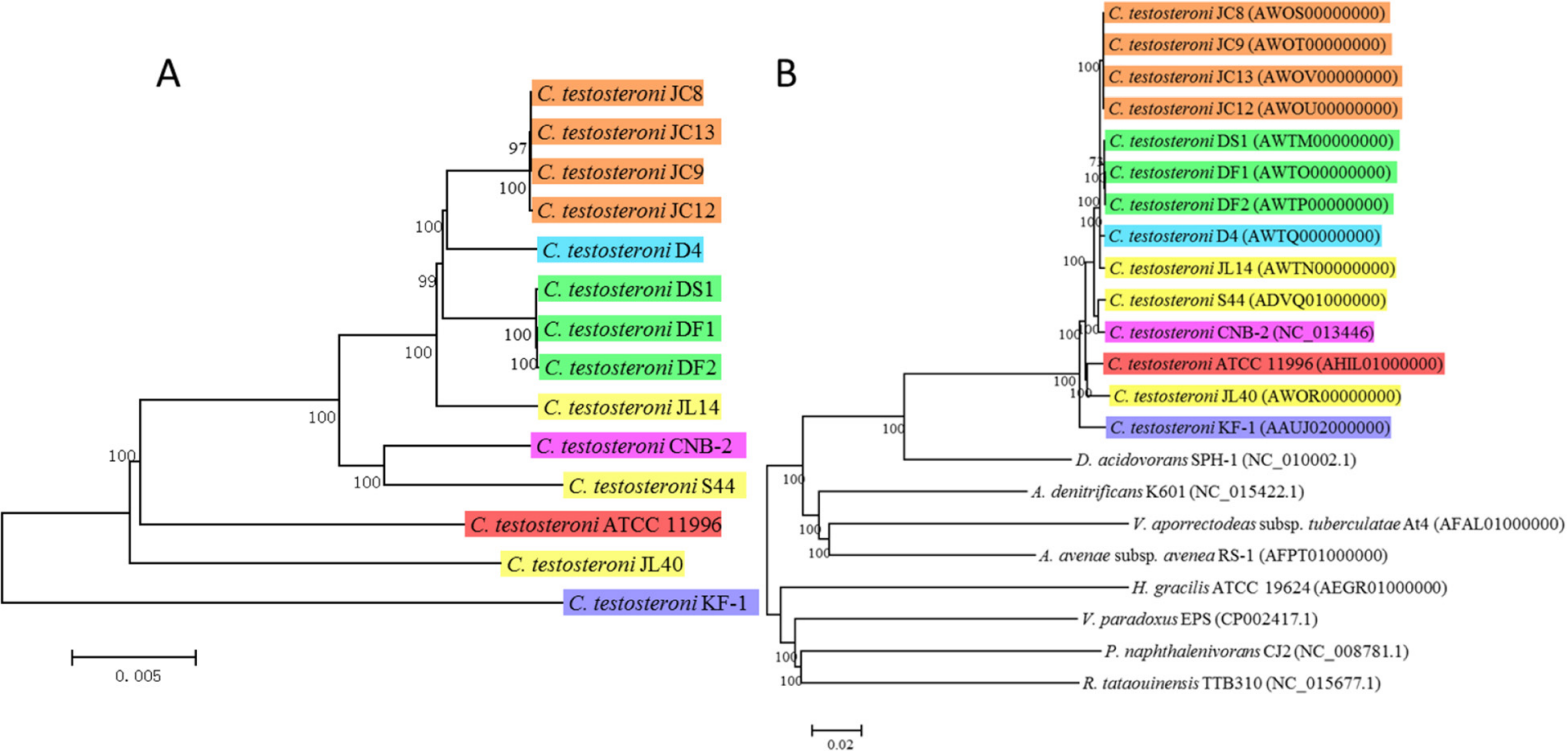
**The NJ phylogenetic trees among 14** ***C. testosteroni*** **strains (A) and among 22 species in** ***Comamonadaceae*** **family (B).** The different colors indicate different sampling areas of the *C. testosteroni* strains as in Figure 1B. The bootstrap-support value before each node represents the confidence degree of each branch. The accession numbers are listed after each strain in Figure B.

To understand the phylogenetic relationship among the *C. testosteroni* strains, a neighbor-joining (NJ) tree based on the core-genome of the 14 *C. testosteroni* strains was built (Figure 2A). The core-genome of the 14 *C. testosteroni* strains contains 3,483 single copy orthologues. As the tree showed, the strains from the same original area are clustered together except for those from Lengshuijiang antimony mine soil. Strains from Lengshuijiang antimony mine (JL14, JL40, and S44) are distributed in different branches. The strain KF-1 is located on a standalone branch.

To understand the phylogenetic relationship between *C. testosteroni* and other species in family *Comamonadacea*, we performed phylogenetic analysis among the 14 *C. testosteroni* strains together with eight more sequenced strains from different species of family *Comamonadacea* (Additional file 2: Table S2). A NJ tree based on a total of 1,003 single copy orthologous gene families of 22 strains was built (Figure 2B). All the 14 *C. testosteroni* strains are grouped together. Strain *Delftia acidovorans* SPH-1, which was classified to genus *Comamonas* before 1999 [52], showed a closer relationship with strains of *C. testosteroni* than with the other seven species of family *Comamonadacea*.

### The relationship between the genome similarity and the geographic distance

**Figure 3:**
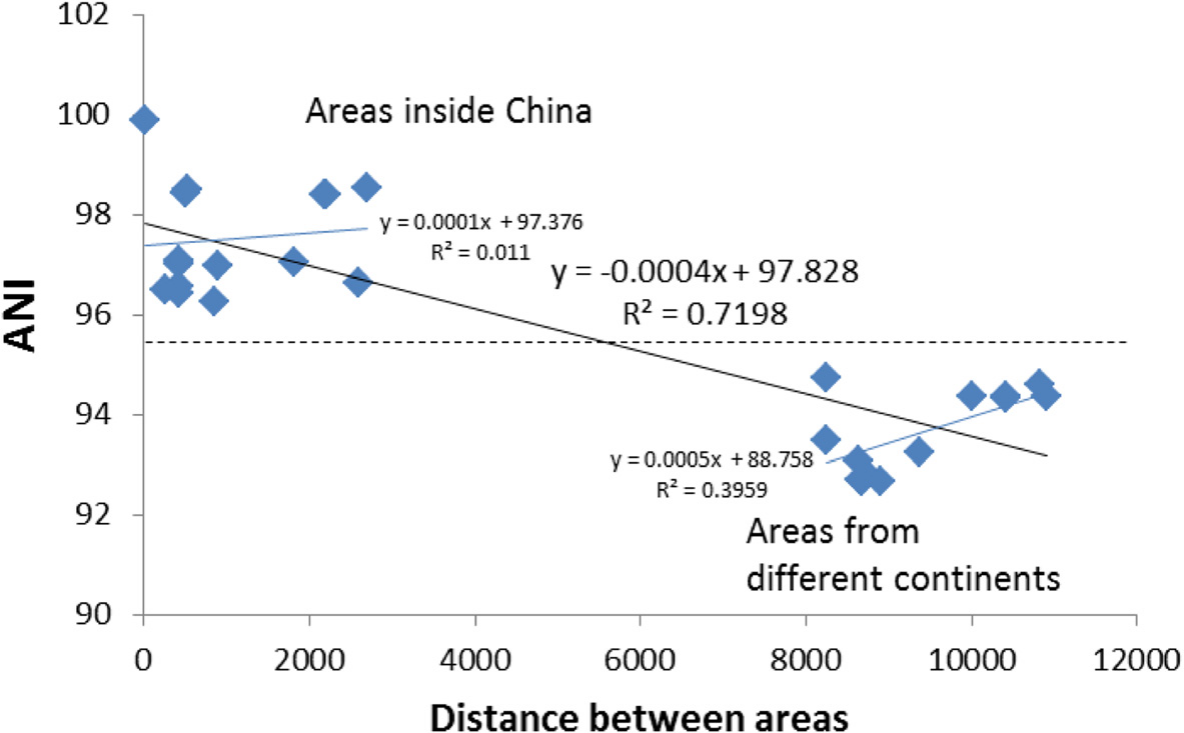
**Distance decay analysis using ANIs among** ***C. testosteroni*** **strains from different areas.** The x axis is the distance between different areas calculated by latitude and longitude (Additional file 1: Table S1). The y axis is the ANI between the pairs of strains isolated from different areas. Linear regressions were taken between the ANIs and distances, and the R squares were given. The dotted line separates the ANI-distance relations among areas inside China (above) and those among areas from different continents.

To understand if there is a distance decay relationship to the genome similarity of this species, we compared the ANIs with the geographic distances among different isolation areas of the *C. testosteroni* strains. As showed in Figure 3, the ANIs for strains from different continents are obviously smaller than for strains from the same continents (isolation areas inside China). However, there is no clear ANI-distance relationship among the isolation areas inside China (R^2^ = 0.011) and from different continents (R^2^ = 0.3959).

### High correlation between phenotypes and genotypes in *C. testosteroni*

**Figure 4:**
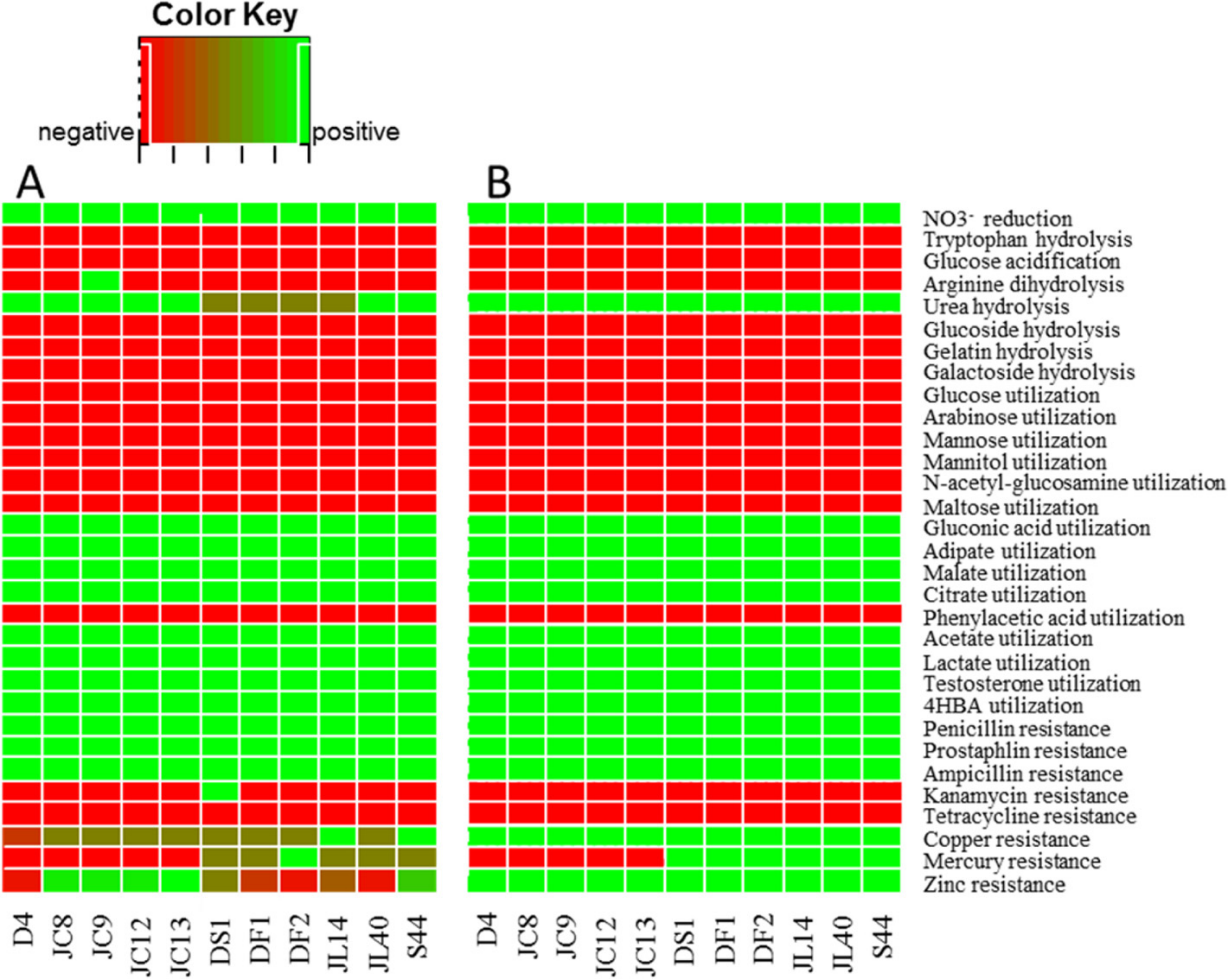
**Correlation between genotypes and phenotypes among the 11** ***C. testosteroni*** **strains. A)** The heat map that presents 31 phenotypes of 11 *C. testosteroni* strains. **B)** The heat map that presents 31 genotypes of 11 *C. testosteroni* strains. The Green/red boxes in the heat map represent positive/negative features of strains. The intermediate color in the row for urea hydrolysis in Figure A means weak positive response, and the intermediate color in the last three rows in Figure A means numerical values.

In order to establish the relationship between phenotypes and genotypes among the strains of *C. testosteroni* and to provide detailed insights into the genomes of this species, 31 important phenotypes and their related genome features of 11 strains of *C. testosteroni* isolated in our laboratory (S44, JL14, JL40, JC8, JC9, JC12, JC13, DS1, D4, DF1, DF2) were tested and studied. Our results showed a high correlation between the phenotypes and genotypes (Figure 4). First, all the trains showed the ability of nitrate reduction and urea hydrolysis; they can utilize testosterone, adipate, malate, gluconate, acetate, lactate and 4HBA as their sole carbon source; they could resist penicillin, prostaphlin, ampicillin, Cu(II) and Zn(II). In the genomes of these strains, we found genes or complete metabolic pathways for these features. Second, none of the strains showed the ability of tryptophan hydrolysis, glucoside hydrolysis, gelatin hydrolysis, galactoside hydrolysis and glucose acidification; they cannot utilize most carbohydrates such as glucose, arabinose, mannose, mannitol, N-Acetyl-D-glucose and maltose; they could not resist tetracycline. In the genomes of these strains, no corresponding genes of these features could be found. Third, the strains DF1, DF2, DS1, S44, JL14, JL40 are resistant and D4, JC8, JC9, JC12, JC13 are sensitive to mercury. In the genomes of DF1, DF2, DS1, S44, JL14, JL40, the mercury resistance genes could be found, but in the genomes of D4, JC8, JC9, JC12 and JC13, the mercury resistance genes could not be found.

In all, we found that 99.4% of a total of 31 × 11 biophysical and biochemical characters were concordant between phenotypes and genotypes. The only two exclusions were that arginine double hydrolysis test showed positive on strain JC9 and kanamycin resistance test showed positive on strain DS1, but the genes for these metabolisms were not found in their genomes.

### Important genotypes of *C. testosteroni*

#### The conservative gene clusters of nitrate/nitrite reduction founded in all the *C. testosteroni* genomes and other genomes of *Comamonadacea*

**Figure 5:**
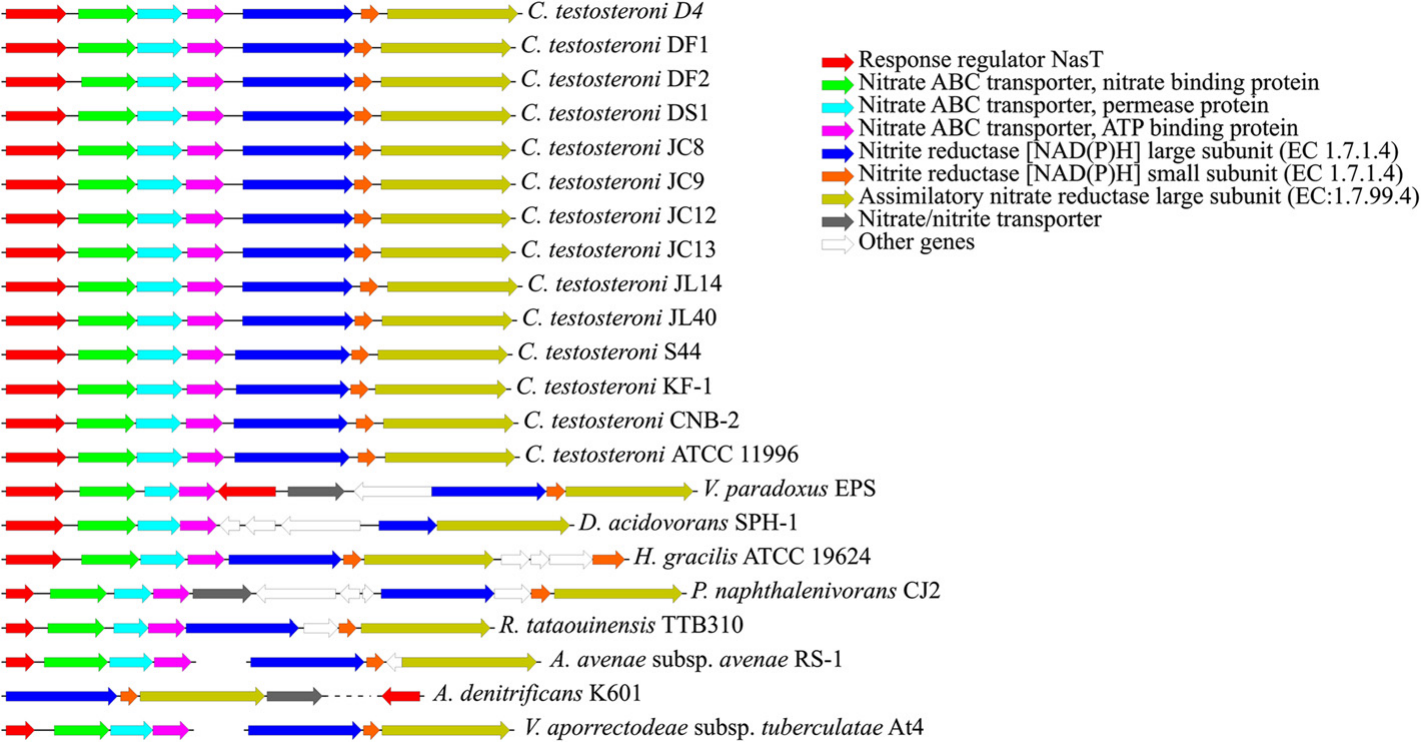
**The nitrate/nitrite reduction gene clusters that were shared by all the** ***C. testosteroni*** **genomes and the eight other genomes of** ***Comamonadacea*** **.** The gene orthologues for different functions are showed in different colors. White arrows indicate genes for other functions that were not correlated with nitrate/nitrite reduction. The breaks in the clusters from *A. avenae* subsp. *avenae* RS-1 and *V. aporrectodeae* subsp. *tuberculatae* At4 mean that the genes are distributed in different contigs. The dotted line in the cluster from *A. denitrificans* K601 means that the interval between the genes is very long.

The physiological and biochemical tests showed that all the strains of *C. testosteroni* have the ability of nitrate reduction. This phenotype has been found in strains of other species of genus *Comamonas* [53]. Based on RAST annotation result, we found 12 gene orthologues in the SEED subsystem “Nitrate and nitrite ammonification”. These genes are distributed in three gene clusters and some dispersal regions in the genomes of *C. testosteroni* (Additional file 5: Table S5). Importantly, a gene cluster responsible for nitrate and nitrite reduction could be found in all of the 14 *C. testosteroni* genomes (Figure 5). Similar gene clusters could also be found in all the eight other genomes of *Comamonadacea*. However, some genes were missed in the cluster, or the gene arrangement was quite different (Figure 5).

#### The key genes for hexose phosphorylation missed in the *C. testosteroni* genomes

**Figure 6:**
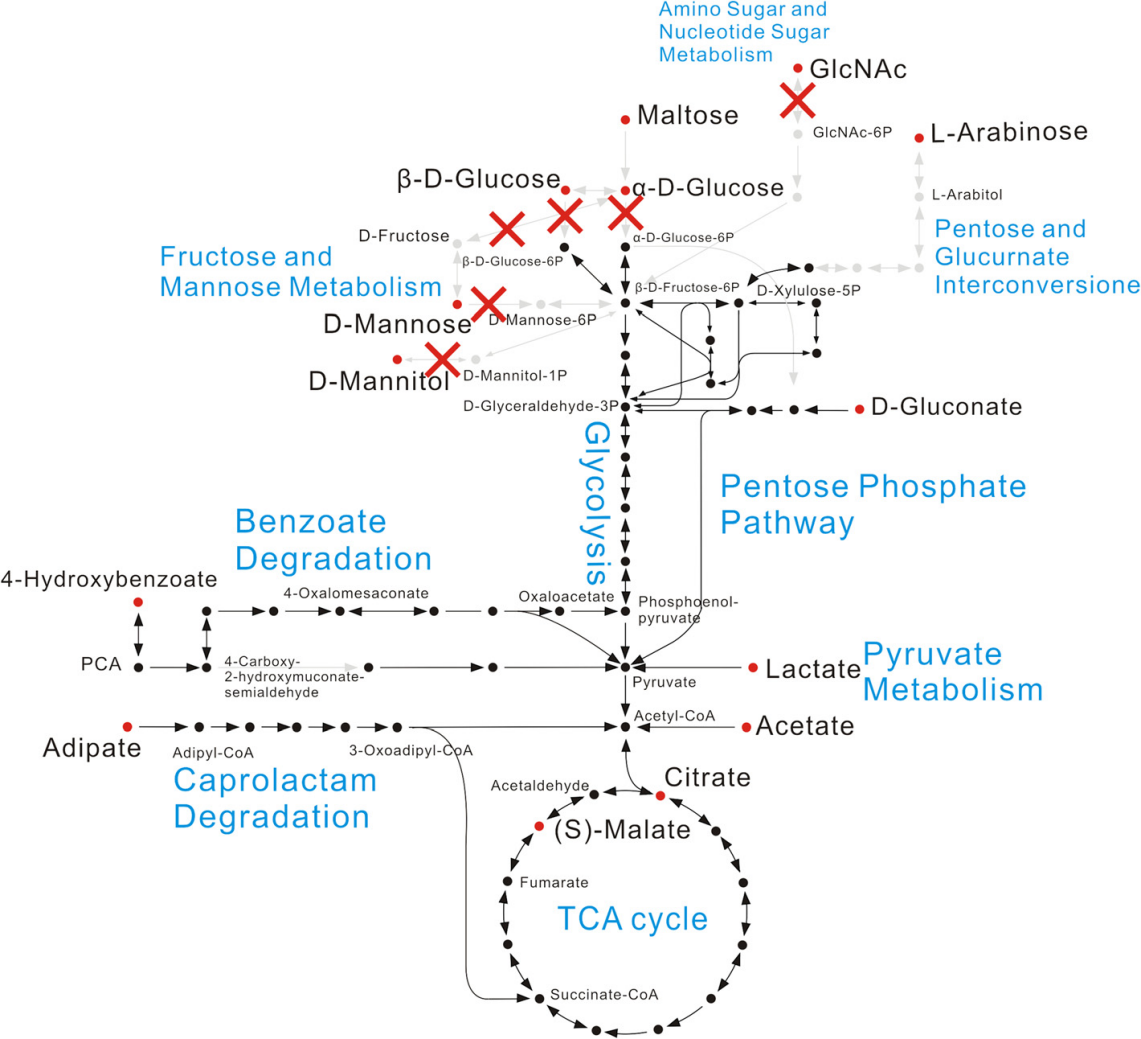
**The assimilatory pathway map of D-glucose, maltose, D-mannose, D-mannitol, D-gluconate, L-arabinose, citrate, malate, acetate, lactate, 4HBA, adipate, and N-acetyl-glucosamine in** ***C. testosteroni*** **.** Labels in blue indicate different pathways of KEGG pathway map. The red points represent the substrates we tested in physiological and biochemical test. Arrows and points in black mean that the pathway could be found whereas those in grey mean that the pathway cannot be found in the genomes of the *C. testosteroni* strains. The red crosses indicate the lacked phosphorylation steps in the utilization procedure of D-glucose, N-Acetyl-D-glucose, D-mannose and D-mannitol. This pathway map is common for all the strains of *C. testosteroni*.

Previous studies [3,5,22] and our phenotype test both indicated that strains of *C. testosteroni* cannot use most types of six carbon sugars as their sole carbon sources. To understand the genome bases of this phenotype, we reconstructed the metabolic pathway map of these strains through Encyclopedia of Genes and Genome (KEGG). The metabolic procedures of the carbon sources in our test (except for decanoic acid, testosterone and phenylacetic acid) were drawn in Figure 6. Complete metabolic pathways of adipate, D-gluconate, citrate, malate, acetate and lactate, which can be utilized by these strains, can be found, whereas the phosphorylation steps in the utilization procedure of D-glucose, N-Acetyl-D-glucose, D-mannose and D-mannitol, which cannot be utilized by these strains, cannot be found. These results are consistent with previous reports that the glucokinase and hexokinase were missed [5,22] in the genomes of *C. testosteroni*.

#### Genomic bases of utilization of steroids and aromatic compounds in *C. testosteroni*

**Figure 7:**
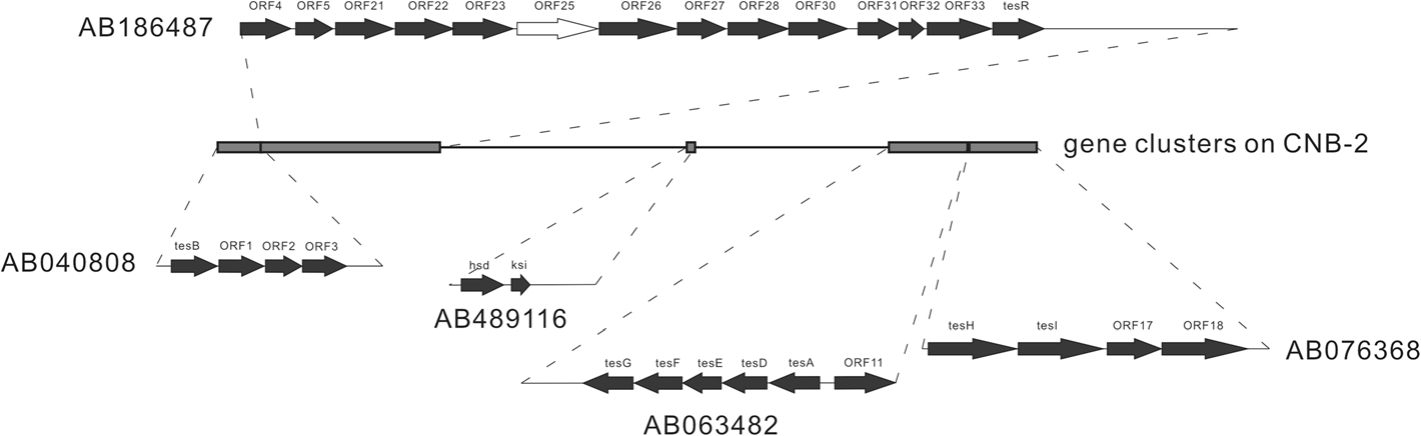
**The structure and distribution of the five gene clusters for steroids degradation on the genomes of the 14** ***C. testosteroni*** **strains.** The distribution of these clusters on the genome of strain CNB-2 is showed in the middle. The genes in each cluster are showed above and below and marked with GenBank accession number of homologous cluster from *C. testosteroni* TA441. The black arrows indicate that the genes could be found in all of the strains. The white arrow indicates that the ORF25 is missed in the strain KF-1 and JL40. The genes of these gene clusters from each genome could be viewed in Additional file 6: Table S6.

Testosterone (a kind of steroids) degradation is the main feature of *C. testosteroni,* based on which this species was identified and named [1]. We searched the five gene clusters relating to steroid degradation, which were found in *C. testosteroni* strain TA441 [15], in the 14 genomes of *C. testosteroni*. As showed in Figure 7, these clusters were distributed in a 105 Kb region on the genome sequence of the strain CNB-2. Based on the genomic DNA sequences, all the five gene clusters (listed in Additional file 6: Table S6) could be found in each of the *C. testosteroni* genomes, while based on the predicted protein sequences, the ORF25 was missed in KF-1 and JL40 genomes (Figure 7). However, the ORF25 is similar to the ORF26 (with 65% identity), and they both showed to have a GGCT-like domain by searching the Conserved Domain Database [54]. Therefore, lacking the ORF25 may not affect the steroid degradation function. We also searched these five gene clusters in other genomes of the family *Comamonadacea* and could not find any complete one. Only the homologous genes of *tes*G and *tes*F could be found (the percent of identity and percent of match are greater than 70%).

**Figure 8:**
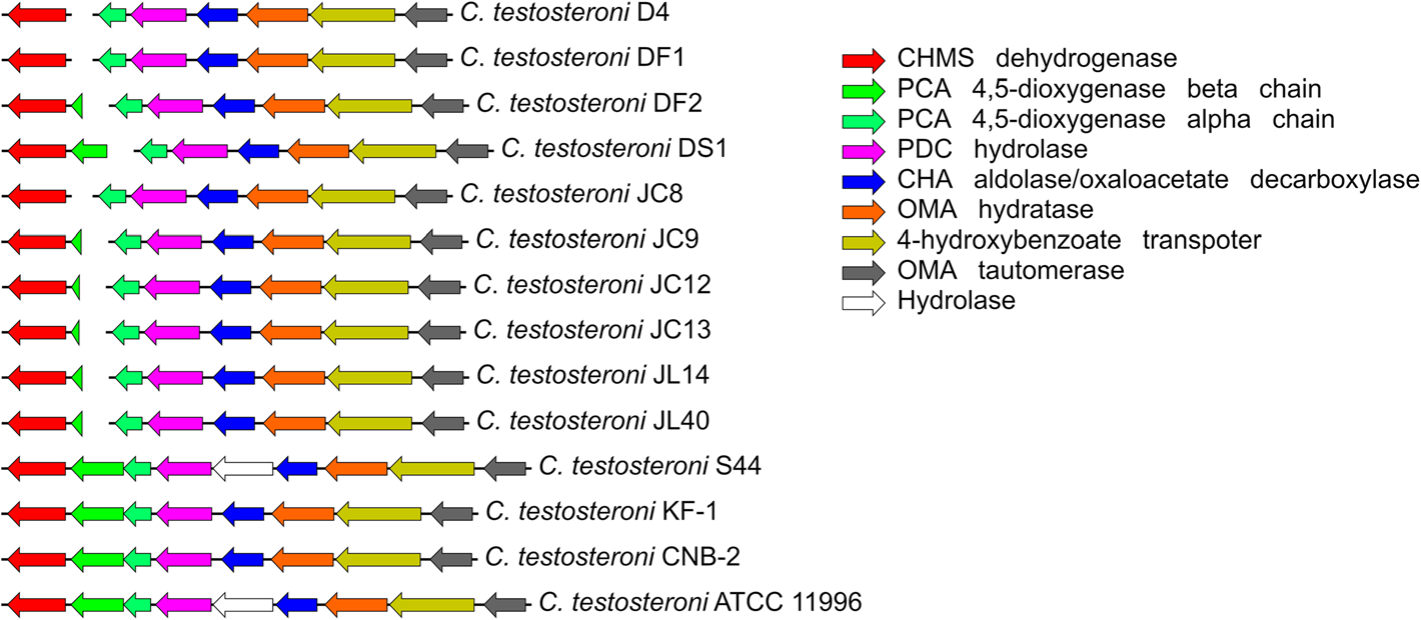
**The gene cluster found in the** ***C. testosteroni*** **genomes that is potentially involved in PCA 4,5-cleavage pathway.** The genes’ functions were annotated according to Ni’s report [20] and RAST annotation result. The orthologues for different functions are showed in different colors. The breaks in the cluster mean that the genes are separated in different contigs. The genes of this cluster from each genome could be viewed in Additional file 7: Table S7.

A previous report indicated that strains of *C. testosteroni* could utilize 4HBA [3]. Our phenotype tests showed that all strains of *C. testosteroni* could use 4HBA as their sole carbon source. We searched the genes for 4HBA degradation using blastp and KEGG annotation tools. The gene cluster which is potentially involved in PCA (protocatechuate) 4,5-cleavage [20], and the gene for transforming 4HBA into PCA were found in the genomes of this species (Figures 6 and 8, Additional file 7: Table S7). This gene cluster was separated into two contigs and part of the ORF that codes for PCA 4,5-dioxygense beta chain was missed in genome sequences of D4, DF1, DF2, DS1, JC8, JC9, JC12, JC13, JL14 and JL40 (Figure 8), probably due to the inability of the illumina sequencing method in this region.

#### The genes for antibiotics and heavy metal resistance

Our previous study showed that the strain S44 of *C. testosteroni* has multiple heavy metal resistances [6]. In this study, we found that all strains of *C. testosteroni* can resist Cu(II), Zn(II), penicillin, prostaphlin and ampicillin. Six of them can resist Hg(II). Based on RAST annotation result, gene clusters and scattered genes relating to heavy metal and medico resistances were found in the genomes of *C. testosteroni*. Of these gene clusters, 11 were shared in all of the 14 genomes (Additional file 8: Table S8). They included homologous genes for RND transporter families such as Czc family of cobalt-zinc-cadmium RND efflux transporter, Cme family of multidrug resistance RND efflux system and multidrug efflux RND membrane fusion protein MexC, and homologous genes for copper homeostasis such as copper homeostasis protein CutE, copper chaperone and copper-translocation P-type ATPase. Besides, there were many types of gene clusters that were not shared in all of these genomes (Additional file 8: Table S8). Genes in these clusters included those resistant to mercury, arsenic and chromium. RND efflux systems of cobalt-zinc-cadmium (Czc family) and multidrug were also found. In addition, many scattered genes such as those for penicillins and fosfomycin resistances were found.

Antibiotics and metal resistances are usually co-selected. The main mechanisms of this phenomenon are co-resistance and cross-resistance [55]. Co-resistance means that the genes for antibiotics and metal resistances are located in the same genetic element. Cross-resistance means that the different resistant phenotypes are controlled by the same gene or the same group of genes. In the genomes of *C. testosteroni*, we found that the genes for different resistance functions were usually clustered in accord with co-resistance. For example, a gene for copper binding protein and a gene for Cu(I) responsive transcriptional regulator are located near some RND efflux Czc family genes (eg. strain D4 Locus Tag: P609_08845 ~ P609_08895 GenBank: AWTQ00000000). Also, the genes for the cross-resistance can be found in these genomes, such as the cobalt-zinc-cadmium efflux system Czc family [56] and multidrug resistance system [57]. Notably, most of these efflux systems belong to the Resistance-Nodulation-cell Division (RND) family.

#### The phylogenetic relationships of the genotypes consistent among the strains of *C. testosteroni*

**Figure 9:**
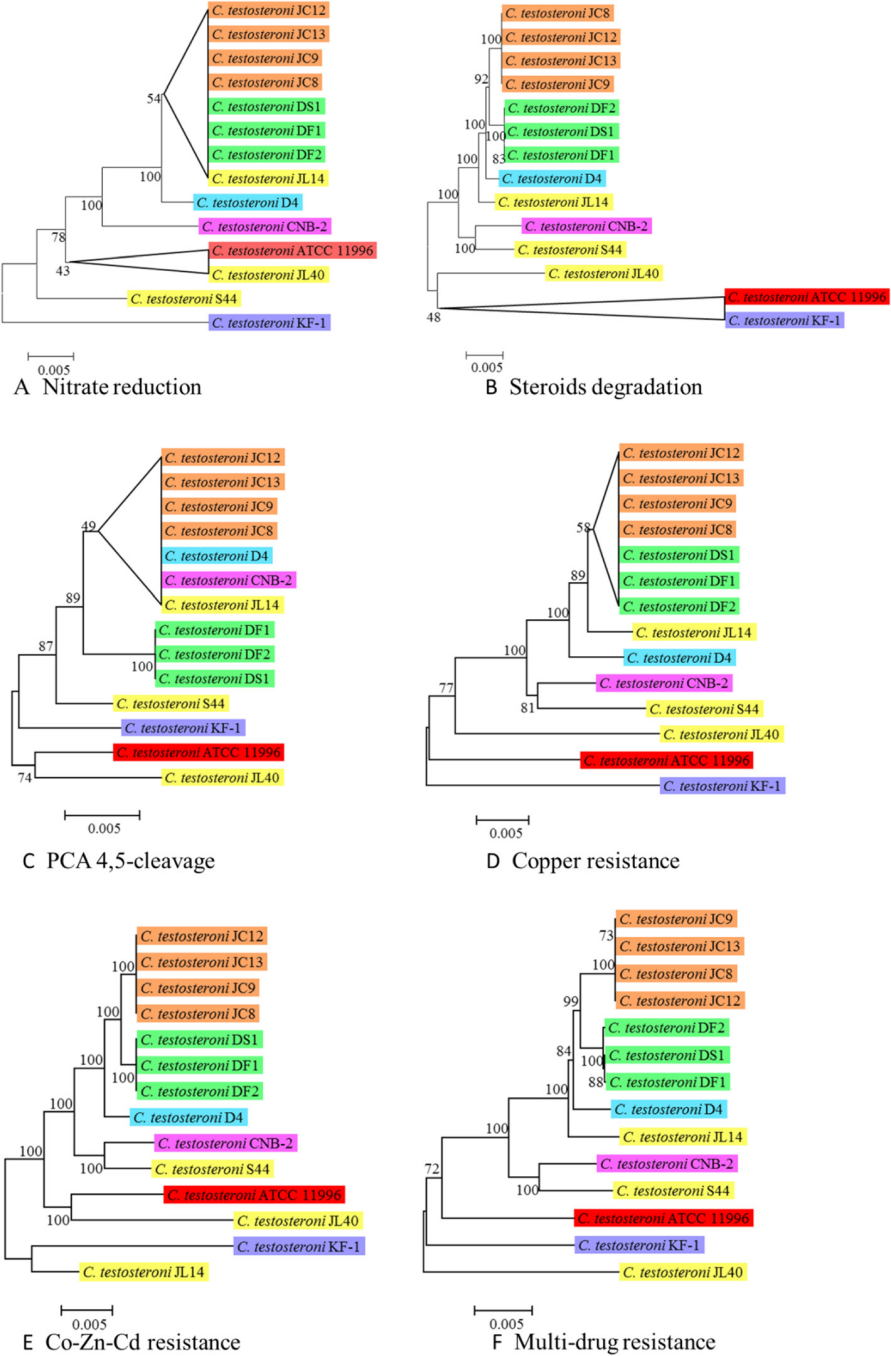
**Phylogenetic analyses of the genes for nitrate reduction, steroids degradation and antibiotics and heavy metal resistance that are consistent among the 14 strains of** ***C. testosteroni*** **. A)** The NJ tree of the genes for nitrate reduction. **B)** The NJ tree of the genes for steroids degradation. **C)** The NJ tree of genes for PCA 4,5-cleavage. **D)** The NJ tree of the genes for copper resistance. **E)** The NJ tree of the genes for cobalt-zinc-cadmium resistance. **F)** The NJ tree of the genes for multidrug resistance. All the genes are from the shared gene clusters. The genes used to build tree A, B and C could be viewed in Additional file 5: Table S5, Additional file 6: Table S6 and Additional file 7: Table S7, respectively. The genes used to build tree D, E and F could be viewed in Additional file 8: Table S8. All trees were built on protein sequences with p-distance. The different colors indicate different sampling areas of the *C. testosteroni* strains as in Figure 1B. The bootstrap-support value before each node represents the confidence degree of each branch. The branches with the bootstrap-support value less than 60 were collapsed.

To analyze the phylogenetic relationships of the genes for nitrate reduction, steroids degradation, PCA 4,5-cleavage and antibiotics and heavy metal resistance that are consistent among the strains of *C. testosteroni*, we built NJ trees of these genes from shared gene clusters (Figure 9). We found that the p-distance between genes of these genotypes are no greater than 0.05. In comparison, the p-distance between homologous proteins from different genus are at least greater than 0.148 (Figure 2B). This indicated that the genes of these clusters are highly conserved. Comparing these trees with the NJ tree of core-genome of the 14 *C. testosteroni* strains (Figure 2A), common features on topology were found. All the strains from Jixi are grouped together and all the strains from Daye are grouped together in each tree. Strains from China (JC8, JC9, JC12, JC13, DF1, DF2, DS1, D4) except those from Lengshuijiang are located closely in all of the trees except that of PCA 4,5-cleavage. The strains from Lengshuijiang are distributed in different branches (JL14, JL40, S44).

### Virulence factors in *C. testosteroni*

In order to identify potential virulence factors (VFs) in the genomes of *C. testosteroni*, we aligned proteins from the 14 genomes against Virulence Factors Data Base (VFDB) [51] using blastp with the standard E-value ≤1e-5, identity ≥ 50%, and match ≥ 70%. As result, we identified 24 types of VFs that were involved in 13 functions such as adherence, anti-phagocytosis, invasion, and secretion system. Of them, 11 VFs are owned by all of the strains. These VFs include 2 types of secretion system (Bsa T3SS, xcp secretion system), urease, flagella of invasion, alginate used for anti-phagocytosis, type IV pili for adherence. Comparing the shared VFs between every two strains, we found that the average identity of the orthologous VFs from different combinations is 99%. The gene numbers of all the found VFs are showed in Additional file 9: Table S9.

## Discussion

The concept of pan-genome was first introduced in 2005 [58]. It is the collection of the total genes that could be found in a species. The pan-genome could reflect the gene pool size of a bacterium [59]. In this study, Heap’s law [40] analysis showed that the pan-genome of *C. testosteroni* is open. However, there are limitations in this analysis. First, the strains were not randomly sampled. Some genomes are very similar (e.g. JC8, JC9, JC12, JC13), and some genomes are significantly different from the others (e.g. KF-1). Therefore, we could notice a “break” in Figure 1A. Second, because most of the genomic sequences are draft, some genes may be lost in the sequences (e.g. in Figure 8, the gene for PCA 4,5-dioxygenase beta chain is lost in some strains due to interruption in the coding region of this gene). This may result in an underestimated core-genome and an overestimated pan-genome.

According to the natural selection theory, environment could affect organisms’ genetic characters. In our study, we found: a) the strains isolated from the same areas (Jixi and Daye) are always clustered together (this indicates the highest similarity), and b) ANIs among strains from different continents are lower than those from the same continents. These results revealed that the genome similarity of most *C. testosteroni* strains followed a certain degree of distance-similarity decay relationship.

In previous studies, strains of *C. testosteroni* showed abilities for nitrate reduction, and utilization of testosterone, 4HBA, acetate and lactate, but they cannot utilize glucose and most of the carbohydrates [3]. In this study, we found that most of the above phenotypes of *C. testosteroni* are consistent with previous studies and consistent among all the tested strains of *C. testosteroni*, indicating that these important phenotypes are very stable and common in *C. testosteroni*, and could be used in the polyphasic taxonomy in identification of this species.

The key step of aromatic compounds degradation is the benzene ring cleavage and the main pathway for this reaction in strains of genus *Comamonas* is PCA 4,5-cleavage [19]. In previous study, genes or potential genes for PCA 4,5-cleavage were found in many strains of *C. testosteroni* such as *C. testosteroni* BR6020 [60] and *C. testosteroni* CNB-2 [20]. In this study, potential genes for this function could be found in all of the genomes of *C. testosteroni* (Additional file 7: Table S7) and the gene cluster for PCA 4,5-cleavage is very conserved among the *C. testosteroni* strains (Figure 9C). This indicated that the strains of *C. testosteroni* would have potential abilities to degrade various aromatic compounds.

Ma *et al.* [5] built a main metabolic pathway map of *C. testosteroni* CNB-2*.* They found that the glycolysis is incomplete due to lack of the hexokinase and glucokinase genes so that the strain cannot utilize most types of sugars. Studies on the genomes of *C. testosteroni* strains ATCC 11996 [22] and KF-1 [23] showed the same results. We built metabolic pathway maps of 14 strains of *C. testosteroni* (including above strains) and showed that all genes for hexose phosphorylation were missed in all genomes of this species (Figure 6). This result effectively supports the previous studies and indicates that lack of these genes should be a common genome feature of *C. testosteroni*.

Comparison between the phylogenomic tree of *C. testosteroni* (Figure 2A) and the phylogenetic trees of the genes from the gene clusters for nitrate/nitrite reduction, steroids degradation, antibiotics and heavy metal resistance (Figure 9A, B, D, E and F) revealed that the gene clusters for these functions are conserved and their evolutions are consistent with the evolution of the core-genome. This indicated that the shared gene clusters for these functions are inherent in the genomes of *C. testosteroni*, and they may have suffered little natural selection pressure during the evolutionary process of *C. testosteroni.*

As an environmental bacterium, *C. testosteroni* is often considered to be nonpathogenic. However, according to the survey by Farshad *et al.* [61], up to 2012, there were 35 reported cases of human infection by this bacterium. *C. testosteroni* infected central nervous system [62], blood [63,64], mitral valve [65], subcutaneous tissue [64], eye [66], abdominal cavity [67] and urinary tract [67], that caused purulent meningitis, bacteremia, cellulitis, infectious endocarditis, post-operative endophthalmitis and other diseases. Nevertheless, few molecular biological investigations were taken on the pathogenicity of *C. testosteroni*. In this study, 24 types of VFs were found in the genomes of *C. testosteroni* with high protein-to-protein identify (≥50%) and match (match ≥70%). Further, we found that the shared VFs among the *C. testosteroni* are highly conserved. This result proposed the molecular biological basis of the potential pathogenicity of this bacterium.

Genotype determines phenotype. Jung *et al.* [68] found many genotypes relating to antibiotic resistance, oxidative stress, motility, and pectin metabolism in *Alishewanella* species, and confirmed the phenotypes of them. Ma *et al.* [5] examined the ability of the utilization of many sugars of *C. testosteroni* CNB-2 and confirmed the genome features that support these phenotypes. However, such a study that focuses on all the main recognition properties and their genome bases on an environmental bacterium is rare. In this study, we found a high correlation between phenotypes and genotypes of this species. The related genes may be useful to study the molecular mechanisms of *C. testosteroni*.

## Conclusions

In conclusion, we generated the sequences of 10 genomes of *C. testosteroni* and performed comparative genomics analysis among the available genomes of this bacterium. We found that the pan-genome of *C. testosteroni* may be open. Physiological and biochemical investigation showed that *C. testosteroni* has the main properties of nitrate reduction, ability of utilizing testosterone, 4HBA, acetate and lactate, and disability of utilizing glucose and most carbohydrates. Genomic analysis revealed the molecular biological bases of these phenotypes. The phenotypes and genotypes of these features could be used in polyphasic taxonomy and molecular identification of this species. This study is the first comprehensive genomic analysis for *C. testosteroni* and provides information for better understanding this bacterial species at genomic level. Importantly, this study focused on the analysis of potential genetic determinants for the typical characters, and found the high correlation between the phenotypes and their corresponding genotypes (e.g. genes)*.*

## Additional files

Additional file 1: Table S1. The sampling areas of the *Comamonas testosteroni* strains. Additional file 2: Table S2. Genomes of the other species from the family *Comamonadacea* that are used in this study. Additional file 3: Table S3. Sequencing results of *Comamonas testosteroni*. Additional file 4: Table S4. The unique genes of each *C. testosteroni* strains that are grouped by OrthoMCL software with inflation value of 1.5. Additional file 5: Table S5. The gene clusters and dispersed genes for nitrate/nitrite reduction in the *C. testosteroni* strains. Additional file 6: Table S6. The gene clusters for steroids degradation in the *C. testosteroni* strains. Additional file 7: Table S7. The gene cluster that is potentially involved in PCA 4,5-cleavage pathway in the *C. testosteroni* strains. Additional file 8: Table S8. The gene clusters and dispersed genes for metal and medico resistance found in *C. testosteroni* strains. Additional file 9: Table S9. Numbers of VFs that are found in genomes of the *C. testosteroni* strains.
