# Supplementary material for: High correlation between genotypes and phenotypes of environmental bacteria Comamonas testosteroni strains
Source: BMC Genomics. 2015 Feb 21;16(1):110. doi: 10.1186/s12864-015-1314-x (PMC4344759; doi:10.1186/s12864-015-1314-x)
Supplement: Additional file 1: Table S1. — The sampling areas of the Comamonas testosteroni strains. [file 12864_2015_1314_MOESM1_ESM.docx]

Table S1. The sampling areas of the *Comamonas testosteroni* strains.

| Strain | Location | Longitude | Latitude | Refference |
| --- | --- | --- | --- | --- |
| *C. testosteroni* JC8 | Coal mine in Jixi of China | 130º57’ E | 45º18’ N | [[1](#_ENREF_1)] |
| *C. testosteroni* JC9 | Coal mine in Jixi of China | 130º57’ E | 45º18’ N | [[1](#_ENREF_1)] |
| *C. testosteroni* JC12 | Coal mine in Jixi of China | 130º57’ E | 45º18’ N | [[1](#_ENREF_1)] |
| *C. testosteroni* JC13 | Coal mine in Jixi of China | 130º57’ E | 45º18’ N | [[1](#_ENREF_1)] |
| *C. testosteroni* JL14 | Sb mine in Lengshuijiang of China | 111º28’ E | 27º45’ N | [[2](#_ENREF_2)] |
| *C. testosteroni* JL40 | Sb mine in Lengshuijiang of China | 111º28’ E | 27º45’ N | [[2](#_ENREF_2)] |
| *C. testosteroni* D4 | Sb polluted soil in Binzhou of China | 113º2’ E | 25º48’ N | This study |
| *C. testosteroni* DS1 | Cu-Fe mine in Daye of China | 114º57’ E | 29º59’ N | [[1](#_ENREF_1)] |
| *C. testosteroni* DF1 | Fe mine in Daye of China | 114º56’ E | 30º12’ N | [[1](#_ENREF_1)] |
| *C. testosteroni* DF2 | Fe mine in Daye of China | 114º56’ E | 30º12’ N | [[1](#_ENREF_1)] |
| *C. testosteroni* S44 | Sb mine in Lengshuijiang of China | 111º28’ E | 27º45’N | [[2](#_ENREF_2)] |
| *C. testosteroni* CNB-2 | Wastewater treatment facility in Nanjing of China | 118º46’ E | 32º2’ N | [[3](#_ENREF_3)] |
| *C. testosteroni* ATCC 11996 | Soil from Berkeley of USA | 122º16’ W | 37º58’ N | [[4](#_ENREF_4)] |
| *C. testosteroni* KF-1 | Lake Konstanz in Germany | 9º11’ E | 47º40’ N | [[5](#_ENREF_5)] |

1. Shi Z, Cao Z, Qin D, Zhu W, Wang Q, Li M, Wang G: **Correlation models between environmental factors and bacterial resistance to antimony and copper.** *PloS one* 2013, **8:**e78533.

2. Li J, Wang Q, Zhang SZ, Qin D, Wang GJ: **Phylogenetic and genome analyses of antimony-oxidizing bacteria isolated from antimony mined soil.** *Int Biodeter Biodegr* 2013, **76:**76-80.

3. Wu JF, Sun CW, Jiang CY, Liu ZP, Liu SJ: **A novel 2-aminophenol 1,6-dioxygenase involved in the degradation of p-chloronitrobenzene by Comamonas strain CNB-1: purification, properties, genetic cloning and expression in Escherichia coli.** *Archives of microbiology* 2005, **183:**1-8.

4. Marcus PI, Talalay P: **Induction and purification of alpha- and beta-hydroxysteroid dehydrogenases.** *The Journal of biological chemistry* 1956, **218:**661-674.

5. Dong W, Eichhorn P, Radajewski S, Schleheck D, Denger K, Knepper TP, Murrell JC, Cook AM: **Parvibaculum lavamentivorans converts linear alkylbenzenesulphonate surfactant to sulphophenylcarboxylates, alpha,beta-unsaturated sulphophenylcarboxylates and sulphophenyldicarboxylates, which are degraded in communities.** *Journal of applied microbiology* 2004, **96:**630-640.
