# Supplementary material for: High correlation between genotypes and phenotypes of environmental bacteria Comamonas testosteroni strains
Source: BMC Genomics. 2015 Feb 21;16(1):110. doi: 10.1186/s12864-015-1314-x (PMC4344759; doi:10.1186/s12864-015-1314-x)
Supplement: Additional file 2: Table S2. — Genomes of the other species from the family Comamonadacea that are used in this study. [file 12864_2015_1314_MOESM2_ESM.docx]

Table S2. Genomes of the other species from the family *Comamonadacea* that are used in this study

| Strain | Size (Mb) | GC(%) | Genome status | | Accession number (reference) |
| --- | --- | --- | --- | --- | --- |
|  |  |  | Draft/finished | Contigs |  |
| *Acidovorax avenae* subsp. *avenae* RS-1 | 5.5 | 68.7 | Draft | 156 | AFPT01000000 [[1](#_ENREF_1)] |
| *Alicycliphilus denitrificans* K601 | 5.0 | 68.0 | Finished | - | NC_015422.1 [[2](#_ENREF_2)] |
| *Delftia acidovorans* SPH-1 | 6.8 | 66.5 | Finished | - | NC_010002.1 [[3](#_ENREF_3)] |
| *Polaromonas naphthalenivorans* CJ2 | 4.4 | 62.5 | Finished | - | NC_008781.1 [[4](#_ENREF_4)] |
| *Ramlibacter tataouinensis* TTB310 | 4.1 | 70.0 | Finished | - | NC_015677.1 [[5](#_ENREF_5)] |
| *Hylemonella gracilis* ATCC 19624 | 3.6 | 65.1 | Draft | 115 | AEGR01000000 [[6](#_ENREF_6)] |
| *Variovorax paradoxus* EPS | 6.6 | 66.5 | Finished | - | CP002417.1 [[7](#_ENREF_7)] |
| *Verminephrobacter aporrectodeae* subsp. *tuberculatae* At4 | 4.7 | 65.6 | Draft | 1082 | AFAL01000000 [[8](#_ENREF_8)] |

1. Xie GL, Zhang GQ, Liu H, Lou MM, Tian WX, Li B, Zhou XP, Zhu B, Jin GL: **Genome sequence of the rice-pathogenic bacterium Acidovorax avenae subsp. avenae RS-1**. *Journal of bacteriology* 2011, **193**(18):5013-5014.

2. Oosterkamp MJ, Veuskens T, Plugge CM, Langenhoff AA, Gerritse J, van Berkel WJ, Pieper DH, Junca H, Goodwin LA, Daligault HE, Bruce DC, Detter JC, Tapia R, Han CS, Land ML, Hauser LJ, Smidt H, Stams AJ: **Genome sequences of Alicycliphilus denitrificans strains BC and K601T**. *Journal of bacteriology* 2011, **193**(18):5028-5029.

3. Schleheck D, Knepper TP, Fischer K, Cook AM: **Mineralization of individual congeners of linear alkylbenzenesulfonate by defined pairs of heterotrophic bacteria**. *Applied and environmental microbiology* 2004, **70**(7):4053-4063.

4. Yagi JM, Sims D, Brettin T, Bruce D, Madsen EL: **The genome of Polaromonas naphthalenivorans strain CJ2, isolated from coal tar-contaminated sediment, reveals physiological and metabolic versatility and evolution through extensive horizontal gene transfer**. *Environmental microbiology* 2009, **11**(9):2253-2270.

5. De Luca G, Barakat M, Ortet P, Fochesato S, Jourlin-Castelli C, Ansaldi M, Py B, Fichant G, Coutinho PM, Voulhoux R, Bastien O, Marechal E, Henrissat B, Quentin Y, Noirot P, Filloux A, Mejean V, DuBow MS, Barras F, Barbe V, Weissenbach J, Mihalcescu I, Vermeglio A, Achouak W, Heulin T: **The cyst-dividing bacterium Ramlibacter tataouinensis TTB310 genome reveals a well-stocked toolbox for adaptation to a desert environment**. *PloS one* 2011, **6**(9):e23784.

6. Chen S, Beeby M, Murphy GE, Leadbetter JR, Hendrixson DR, Briegel A, Li Z, Shi J, Tocheva EI, Muller A, Dobro MJ, Jensen GJ: **Structural diversity of bacterial flagellar motors**. *The EMBO journal* 2011, **30**(14):2972-2981.

7. Han JI, Spain JC, Leadbetter JR, Ovchinnikova G, Goodwin LA, Han CS, Woyke T, Davenport KW, Orwin PM: **Genome of the Root-Associated Plant Growth-Promoting Bacterium Variovorax paradoxus Strain EPS**. *Genome announcements* 2013, **1**(5).

8. Kjeldsen KU, Bataillon T, Pinel N, De Mita S, Lund MB, Panitz F, Bendixen C, Stahl DA, Schramm A: **Purifying selection and molecular adaptation in the genome of Verminephrobacter, the heritable symbiotic bacteria of earthworms**. *Genome biology and evolution* 2012, **4**(3):307-315.
