# Supplementary material for: High correlation between genotypes and phenotypes of environmental bacteria Comamonas testosteroni strains
Source: BMC Genomics. 2015 Feb 21;16(1):110. doi: 10.1186/s12864-015-1314-x (PMC4344759; doi:10.1186/s12864-015-1314-x)
Supplement: Additional file 3: Table S3. — Sequencing results of Comamonas testosteroni. [file 12864_2015_1314_MOESM3_ESM.docx]

Table S3. Sequencing results of *Comamonas testosteroni*.

| Strain | Sequencing platform | Statistics of high quality data* | | | | |  | Statistics of assembly results | | | | |
| --- | --- | --- | --- | --- | --- | --- | --- | --- | --- | --- | --- | --- |
|  |  | Paired reads number | Single reads number | Average read length (bp) | Total bases (bp) | Coverage |  | Number of contigs | Bases of all contigs | Contig N50 (bp) | Contig N90 (bp) | Largest contig (bp) |
| *C. testosteroni* JC8 | Illumina | 3,835,964 x 2 | 214,506 | 94 | 744,071,662 | 138.9x |  | 123 | 5,356,532 | 143,663 | 47,039 | 578,086 |
| *C. testosteroni* JC9 | Illumina | 5,069,028 x 2 | 147,354 | 96 | 983,963,651 | 183.2x |  | 106 | 5,371,351 | 201,281 | 52,027 | 635,416 |
| *C. testosteroni* JC12 | Illumina | 5,023,066 x 2 | 153,230 | 96 | 974,916,331 | 182x |  | 94 | 5,357,472 | 201,237 | 47,051 | 578,285 |
| *C. testosteroni* JC13 | Illumina | 4,468,524 x 2 | 232,592 | 95 | 867,197,081 | 162.8x |  | 101 | 5,326,602 | 201,237 | 49,513 | 578,285 |
| *C. testosteroni* JL14 | Illumina | 5,978,464 x 2 | 383,694 | 95 | 1,169,010,166 | 224x |  | 125 | 5,746,174 | 136,825 | 25,928 | 499,904 |
| *C. testosteroni* JL40 | Illumina | 3,565,354 x 2 | 200,955 | 94 | 692,065,555 | 116.2x |  | 162 | 5,956,604 | 201,703 | 50,369 | 858,069 |
| *C. testosteroni* D4 | Illumina | 7,161,094 x 2 | 244,223 | 94 | 1,373,303,176 | 271.3x |  | 75 | 5,061,365 | 242,732 | 56,606 | 506,289 |
| *C. testosteroni* DS1 | Illumina | 8,397,390 x 2 | 279,645 | 94 | 1,610,210,738 | 283.2x |  | 158 | 5,686,269 | 114,867 | 29,585 | 447,832 |
| *C. testosteroni* DF1 | Illumina | 7,892,385 x 2 | 272,245 | 94 | 1,514,260,712 | 271.1x |  | 238 | 5,586,485 | 98,152 | 18,558 | 439,132 |
| *C. testosteroni* DF2 | Illumina | 6,983,486 x 2 | 262,184 | 94 | 1,338,119,746 | 238.6x |  | 193 | 5,608,755 | 111,677 | 34,437 | 349,082 |

*The high quality data was obtained from the raw data by masking the adapter sequences, wiping off the bases on the 5’ ends that are not “A, T, C, G”, removing the low quality read ends (Quality<20), then dislodging the reads whose “N” contents are greater than 10%, and finally abandoning the reads whose lengths are shorter than 25 bp.
