# Supplementary material for: High correlation between genotypes and phenotypes of environmental bacteria Comamonas testosteroni strains
Source: BMC Genomics. 2015 Feb 21;16(1):110. doi: 10.1186/s12864-015-1314-x (PMC4344759; doi:10.1186/s12864-015-1314-x)
Supplement: Additional file 9: Table S9. — Numbers of VFs that are found in genomes of the C. testosteroni strains. [file 12864_2015_1314_MOESM9_ESM.docx]

Table S9. Numbers of VFs that are found in genomes of the *C. testosteroni* strains.

| **VF function** | **VFs** | **CNB-2** | **KF-1** | **S44** | **ATCC 111996** | **JC8** | **JC9** | **JC12** | **JC13** | **JL14** | **JL40** | **DF1** | **DF2** | **DS1** | **D4** |
| --- | --- | --- | --- | --- | --- | --- | --- | --- | --- | --- | --- | --- | --- | --- | --- |
| Adherernce | Flagella | - | - | - | 4 | - | - | - | - | - | - | - | - | - | - |
|  | Hsp60 | 1 | 1 | 1 | 1 | 1 | 1 | 1 | 1 | 1 | 1 |  | 1 | 1 | 1 |
|  | Type IV pili | 7 | 7 | 7 | 6 | 7 | 7 | 7 | 7 | 6 | 7 | 6 | 6 | 6 | 6 |
| Ant-iphagocytosis | Capsule | 1 | 2 | - | - | 2 | 2 | 1 | 1 | 1 | 2 | - | - | - | - |
|  | Hyaluronic acid capsule | 2 | 1 | 1 | - | - | - | - | - | - | 1 | - | - | - | - |
|  | Alginate | 1 | 1 | 1 | 1 | 1 | 1 | 1 | 1 | 1 | 1 | 1 | 1 | 1 | 1 |
| Cellular metabolism | Isocitrate lyase | 1 | 1 | 1 | 1 | 1 | 1 | 1 | 1 | 1 | 1 |  |  | 1 | 1 |
|  | PanC/PanD | 1 | 1 | 1 | 1 | 1 | 1 | 1 | 1 | 1 | 1 | 1 | 1 | 1 | 1 |
| Endotoxin | LPS | - | - | - | - | 2 | 2 | 2 | 2 | - | - | - | - | - | - |
| Enzyme | Urease | 2 | 2 | 2 | 2 | 2 | 2 | 2 | 2 | 2 | 2 | 2 | 2 | 2 | 2 |
| Immune evasion | Vi antigen | - | - | - | 2 | 1 | 1 | 1 | 1 | - | - | - | - | - | - |
| Invasion | Flagella | 15 | 15 | 15 | 15 | 15 | 15 | 15 | 15 | 15 | 15 | 15 | 15 | 15 | 14 |
|  | K1 capsule | - | - | - | - | - | - | - | - | - | - | - | - | - | 1 |
| Iron uptake | Enterobactin | 1 | - | 1 | - | 1 | 1 | 1 | 1 | 1 | - | 1 | 1 | 1 | 1 |
|  | Pyoverdine | 1 | - | 1 | - | - | - | - | - | 1 | - | 1 | 1 | 1 | 1 |
| Magnesium uptake | MgtBC | 1 | 2 | 1 | 1 | 1 | 1 | 1 | 1 | 1 | 1 | 1 | 1 | 1 | 1 |
| Regulation | Fur | 1 | 1 | 1 | 1 | 1 | 1 | 1 | 1 | 1 | 1 | 1 | 1 | 1 | 1 |
| Secretion system | Bsa T3SS | 1 | 1 | 1 | 1 | 1 | 1 | 1 | 1 | 3 | 2 | 1 | 1 | 2 |  |
|  | Flagella | 2 | 2 | 2 | 2 | 2 | 2 | 2 | 2 | 2 | 2 | 2 | 2 | 2 | 2 |
|  | xcp secretion system | 2 | 2 | 2 | 2 | 2 | 2 | 2 | 2 | 2 | 2 | 2 | 2 | 2 | 2 |
| Stress protein | KatAB | 1 | 1 | 1 | 1 | 1 | 1 | 1 | 1 | 1 | 1 | 1 | 1 | 1 | 1 |
|  | ClpP | 1 | 2 | 2 | 1 | 1 | 1 | 1 | 1 | 1 | 1 | 1 | 1 | 1 |  |
|  | SodB | 1 | 1 | 1 | 1 | 1 | 1 | 1 | 1 | 1 | 1 | 1 | 1 | 1 | 1 |
| Unclassified | O-antigen | 1 | 1 | 1 | 1 | 1 | 1 | 1 | 1 | 1 | 3 | 1 | 1 | 1 | 1 |
